# Dehydrated Hereditary Stomatocytosis (DHS): A Rare Inherited Hemolytic Disorder With Unusual Hypochromic Microcytic Anemia

**DOI:** 10.7759/cureus.81335

**Published:** 2025-03-28

**Authors:** Badriah G Alasmari, Shady Wafa, Amjad Alsari Alqahtani, Bahaa Sameh, Lina Elzubair

**Affiliations:** 1 Pediatrics, Armed Forces Hospital Southern Region, Khamis Mushait, SAU; 2 College of Medicine, King Khalid University, Abha, SAU; 3 Pathology, Armed Forces Hospital Southern Region, Khamis Mushait, SAU

**Keywords:** dehydrated hereditary stomatocytosis, microcytic hypochromic anemia, non-immune hemolytic disorder, piezo1 mutations, pseudohyperkalemia

## Abstract

Dehydrated hereditary stomatocytosis (DHS) is an autosomal dominant (AD), non-immune hemolytic disorder due to increased erythrocyte membrane cation permeability that leads to red blood cell (RBC) dehydration and lysis, which can present with a wide range of clinical findings. DHS can be present with silent-to-mild normocytic or macrocytic anemia, increased risk of thrombotic complications, or partially compensated hemolysis with few symptoms. Senicapoc has been used recently to treat DHS as it showed activity against RBC dehydration in vitro; however, its clinical outcome is not established. In this study, we report an unusual case of a 10-year-old male child who was misdiagnosed with iron deficiency anemia (IDA) for three years, despite persistent anemia and unresponsiveness to iron therapy. The diagnosis was done using whole exome sequencing (WES).

## Introduction

The human erythrocyte membrane is a laminated structure of an outer lipid bilayer and a two-dimensional network of spectrin-based cytoskeleton. Disorders of the red cell cytoskeleton can be presented with hereditary spherocytosis (HS), hereditary pyropoikilocytosis (HPP), and hereditary elliptocytosis (HE). Disorders of cation cell wall membrane permeability are represented by hereditary stomatocytosis [1]. Dehydrated hereditary stomatocytosis (DHS) is also known as hereditary xerocytosis and is a rare autosomal dominant (AD) hereditary red cell membrane disorder characterized by RBC membranes' permeability to cations. DHS prevalence rate is unknown and can be estimated at 1:50,000 [2]. DHS can be classified into two subgroups as follows: either syndromic with extra-hematological manifestations like anemia, pseudohyperkalemia, and prenatal and/or perinatal edema, or non-syndromic with isolated erythroid phenotype [2]. Recently, PIEZOs were identified as mechanically activated (MA) cation channels; they are expressed in many cell types, including human erythroid, and they act as a sensor of cell tension. PIEZO1 mutations can cause both syndromic and non-syndromic forms of DHS [3]. Individuals with pathogenic variants in PIEZO1 have the risk of severe complications, including thromboembolic events and pulmonary hypertension after splenectomy, so it is not recommended for patients with PIEZO1 mutations. Manifestations of DHS vary from asymptomatic to severe forms of anemia, pallor, fatigue, jaundice, gallstones, splenomegaly, and severe iron overload [4]. Generally, DHS presented with compensated hemolytic anemia, high reticulocyte count, macrocytosis, and mild jaundice. RBC cell wall dehydration results from the loss of the cation content with a subsequent increase of mean corpuscular hemoglobin concentration (MCHC) (>36 g/dL). A blood smear may show the stomatocytes and erythrocytes with a characteristic central mouth-shaped spot, which is quite rare that making diagnosis difficult [5]. Osmotic gradient ektacytometry is a useful and often critical examination to diagnose this condition. It shows a leftward shift of the osmolarity curve due to the presence of dehydrated erythrocytes [6]. Usually, DHS is misdiagnosed or undiagnosed with other conditions, especially hereditary spherocytosis, and sometimes the diagnosis is delayed till adulthood. Here, we report a new case of a 10-year-old male patient from the southern region of Saudi Arabia, initially misdiagnosed with iron deficiency anemia (IDA) and later diagnosed with non-syndromic DHS using whole exome sequencing (WES).

## Case presentation

**Figure 1:**
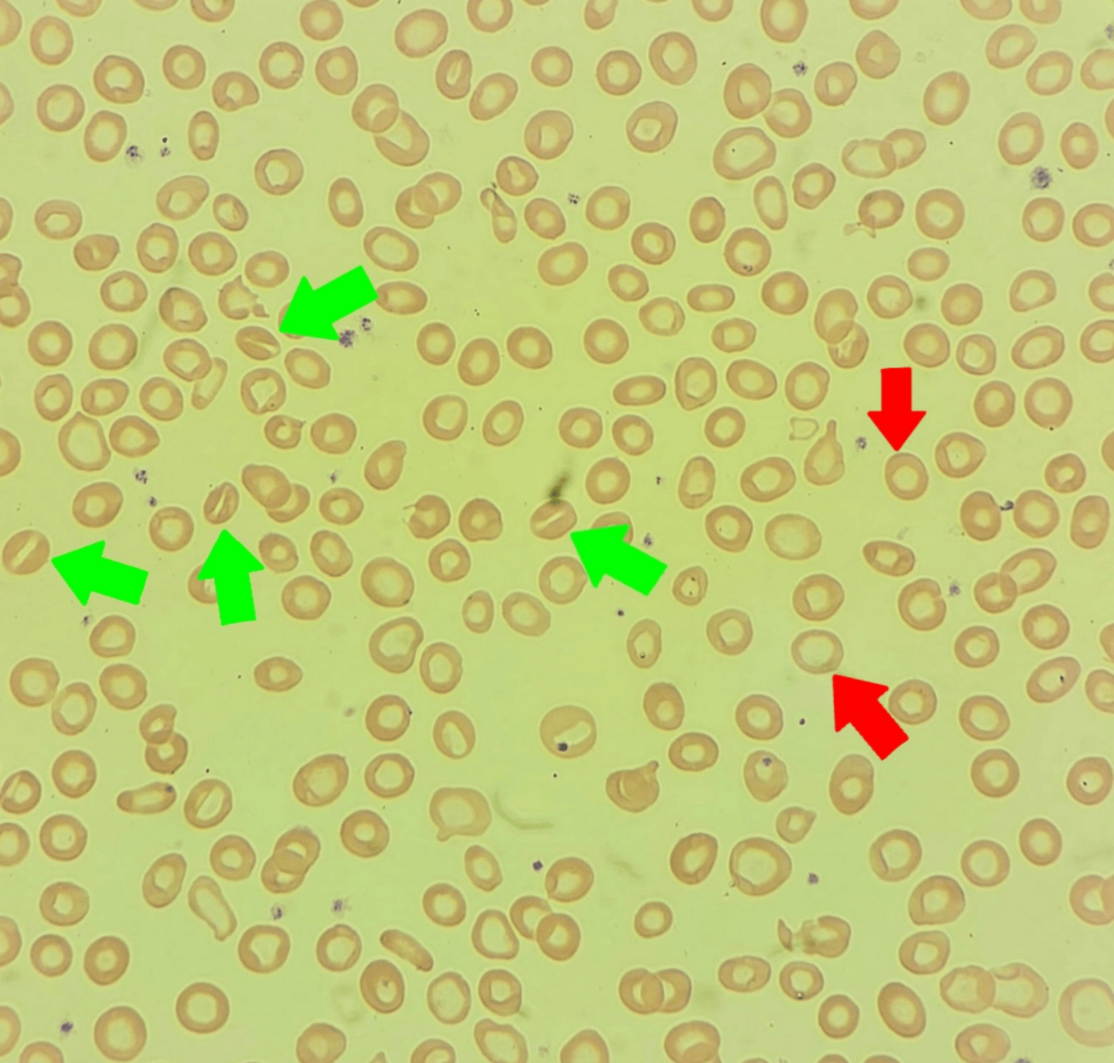
Peripheral blood smear with Wright-Giemsa stain. The images show stomatocytes (green arrows) and hypochromic microcytic RBC (red arrows).

We present a case of a 10-year-old male patient from the southern region of Saudi Arabia who initially presented at the age of three years to the primary clinic with pallor, easy fatigability, and limb weakness. A full blood workup was done and showed hypochromic microcytic anemia, which was diagnosed at this time as iron deficiency anemia (IDA). So, the patient started on oral iron therapy with regular follow-up till the age of six years. During this period, the patient always presented with persistent hypochromic microcytic anemia. At the age of seven, the patient was severely anemic and received blood transfusions three times during that year. Afterward, the patient resumed oral iron therapy. At the age of 10 years, the patient was referred from the family medicine clinic to our hematology clinic for further evaluation. A good history review with careful examination of this patient showed that there was no history of jaundice, dark urine, significant bleeding, ecchymosis, or headache. His family history showed that his parents as well as his brother had the same presentation, and the rest of his family was negative for hemolytic disorders or thrombosis. Initial laboratory workup showed hypochromic microcytic anemia with low serum ferritin and iron (Table 1). A peripheral blood smear test was done and showed hypochromic microcytic RBCs with few stomatocytes (Figure 1). All of this finding raises our suspicion of different types of non-immune hemolytic anemias. So, whole exome sequencing (WES) was done, and the PIEZO1 gene mutation was detected, confirming DHS. The iron therapy was discontinued and we started our management by observation of hemolysis every three months by hemolytic parameters (reticulocyte count, lactate dehydrogenase {LDH}, elevated unconjugated bilirubin), folic acid support, monitoring of iron overload, gallstone symptoms, and close monitoring for thrombosis.

**Table 1:** Recent labs done during last follow-up. Hb: hemoglobin; MCV: mean corpuscular volume; MCH: mean corpuscular hemoglobin; MCHC: mean corpuscular hemoglobin concentration; HCT: hematocrit; RDW: red cell distribution width; LDH: lactate dehydrogenase; TIBC: total iron binding capacity

Lab test	Lab result	Reference range
Hb	7.28 g/dL	10.9-15 g/dL
MCV	55 fL	73-89 fL
MCH	17.8 pg	23-30 pg
MCHC	32 g/dL	32-36 g/dL
HCT	22.5%	31-41%
RDW	20%	11-14%
Reticulocyte count	1.35%	0.5-2.5%
Serum ferritin	3.2 ng/mL	10.3-55.8 ng/mL
LDH	415 IU/L	155-290 IU/L
Serum iron	2.1 umol/L	12.5-32.2 umol/L
TIBC	64.60 umol/L	30-90 umol/L
Transferrin saturation	3.25%	<50%

## Discussion

DHS is an autosomal dominant (AD), non-immune hemolytic disorder caused by PIEZO1 mutations that lead to abnormal cation permeability, RBC dehydration, and chronic hemolysis. PIEZO1 mutations can cause both syndromic and non-syndromic forms of DHS [3]. We report a case of a 10-year-old male patient with non-syndromic DHS, who was misdiagnosed as IDA and received multiple courses of oral iron supplementation without improvement. At the age of three years, our patient started to develop limb weakness and fatigability, started on iron therapy for four years with no improvement, and then at the age of seven years, he received multiple blood transfusions. Our patient's laboratory follow-up consistently showed negative hemolytic parameters, with serum iron and ferritin levels always low, a normal reticulocyte count, and hypochromic microcytic anemia, all of which were inconsistent with chronic hemolysis. At the age of 10, WES was performed, and the patient was diagnosed with DHS, presenting unusually with hypochromic microcytic anemia and no signs of chronic hemolytic anemia, as the patient never complained of jaundice, dark urine, gallstones, or splenomegaly. Another study reported an adult Asian female who was not diagnosed at the age of 39 when she presented with fatigue and was evaluated for anemia. Her evaluation revealed hemoglobin at 8.2 g/dL, iron saturation at 85%, and ferritin at 1961.4 ng/mL. Her peripheral blood smear showed anisopoikilocytosis, macrocytes, spherocytes, and several stomatocytes along with polychromatophils. Based on these findings, a diagnosis of DHS was suspected, and genetic testing was performed, which revealed a mutation in the PIEZO1 [7]. A case study reported an 11-year-old male who was followed due to chronic non-immune, hemolytic anemia with no definitive diagnosis since the age of one year. This patient has a history of a similar condition to his brother and complains of jaundice, pallor, and splenomegaly. This patient was not diagnosed till the age of 11 years when he underwent a full anemia evaluation showing normochromic normocytic anemia and his peripheral blood smear revealed severe polychromasia, anisocytosis with stomatocytes. Diagnosis with DHS due to PIEZO1 gene mutation was done using the Sanger method [2]. Comparing our patient with other reported cases, he was presented with only hypochromic microcytic anemia with no manifestations of hemolytic anemia, which made the diagnosis very challenging and not easy to reach. This case shows the importance of reconsidering the differential diagnosis in any child presenting with anemia that is unresponsive to appropriate iron therapy, especially when supported by normal or borderline iron studies and laboratory evidence of hemolysis. Moreover, this case highlights the crucial diagnostic role of advanced laboratory testing, such as genetic testing and ektacytometry, which can identify abnormal red cell deformability consistent with DHS and provide definitive confirmation of the underlying PIEZO1 mutation.

## Conclusions

DHS is usually misdiagnosed or undiagnosed as other conditions. So, it should be considered a differential diagnosis in patients with refractory anemia who are not responding to iron therapy. Early diagnosis is important in preventing severe complications of iron overload, which leads to multiorgan damage. Genetic testing provides definitive confirmation of the underlying PIEZO1 mutation. Recognizing DHS early allows for targeted management strategies, including folic acid supplementation, monitoring for hemolytic and thrombotic complications, and patient and family education regarding avoiding splenectomy, as it carries a significantly increased risk of life-threatening thrombotic complications.
